# Supplementary figures and images for: A global sensitivity analysis approach for morphogenesis models
Source: BMC Syst Biol. 2015 Nov 21;9:85. doi: 10.1186/s12918-015-0222-7 (PMC4654849; doi:10.1186/s12918-015-0222-7)

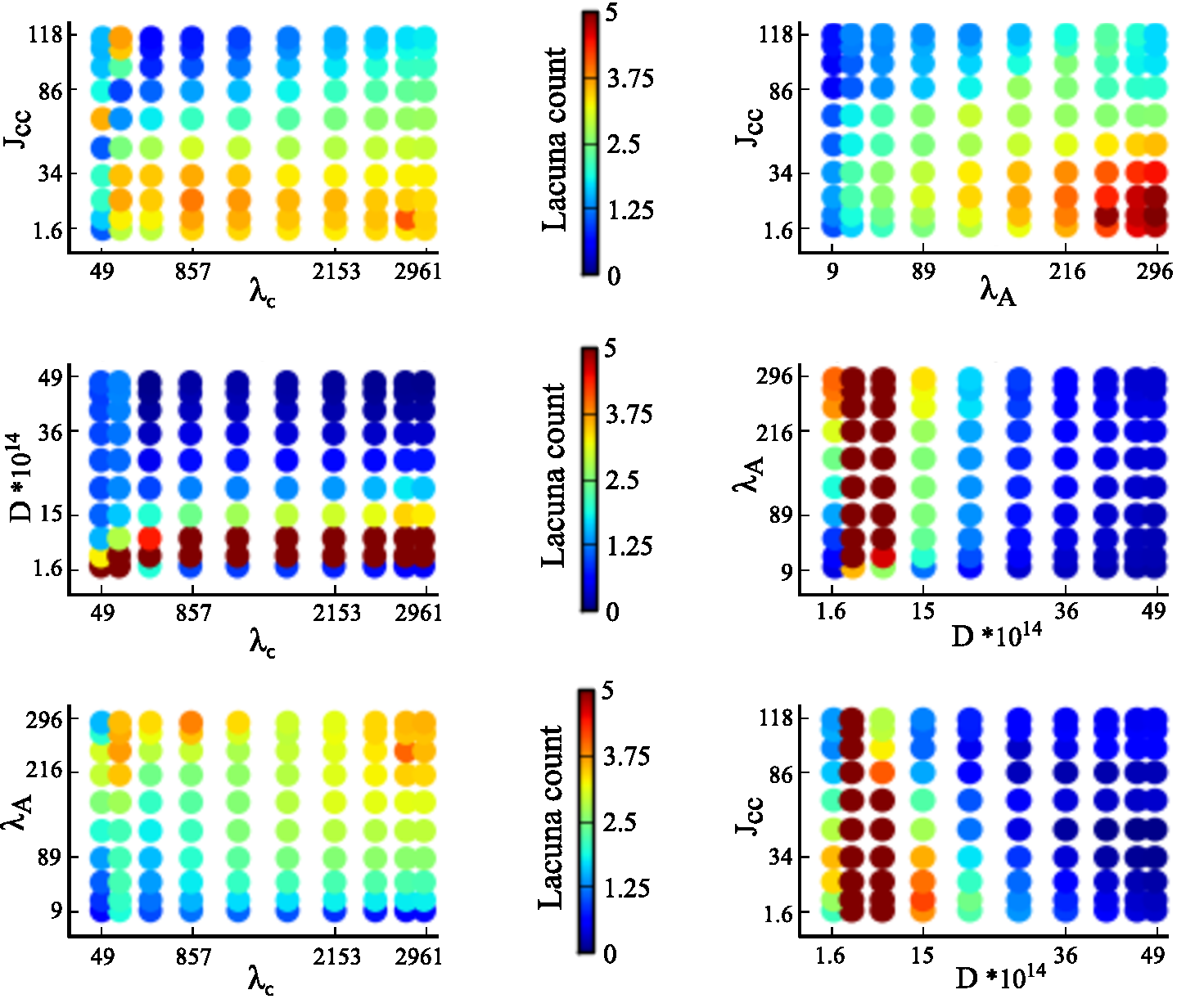

Supplement: Additional file 1 — Figure S1. Two-dimensional intensity plots of lacuna count with maximum intensity of 5 lacunae. The intensity of the output measure lacuna count, mapped to an interval of 0 to 5 as indicated by the color bars, is plotted for each two-parameter combination of the parameters the cell rigidity (λ A), cell-cell adhesion (J cell,cell), the diffusion coefficient of the chemoattractant (D), and sensitivity of cells to the chemoattractant at cell-matrix interfaces (λ c). (PNG 568 Kb) [file 12918_2015_222_MOESM1_ESM.png]
