# Supplementary material for: A global sensitivity analysis approach for morphogenesis models
Source: BMC Syst Biol. 2015 Nov 21;9:85. doi: 10.1186/s12918-015-0222-7 (PMC4654849; doi:10.1186/s12918-015-0222-7)
Supplement: Additional file 2 — Table S1. Global sensitivity analysis results for compactness. (PDF 51.7 Kb) [file 12918_2015_222_MOESM2_ESM.pdf]

Table S1 Global sensitivity analysis results for compactness.

$\hat{p}$	12	13	14	15
Variance data	0.0453	0.0453	0.0453	0.0453
Variance PCE	0.0452	0.0453	0.0454	0.0456
S(λ_c)	0.3190	0.3188	0.3186	0.3183
S(D)	0.2971	0.2969	0.2965	0.2958
S(λ_A)	0.0267	0.0266	0.0266	0.0265
S($J_{\text{cell,cell}}$)	0.2052	0.2048	0.2043	0.2032
S(λ_c,D)	0.0124	0.0125	0.0126	0.0127
S(λ_c,λ_A)	0.0107	0.0107	0.0109	0.0110
S(λ_c,$J_{\text{cell,cell}}$)	0.0558	0.0559	0.0561	0.0570
S(D,λ_A)	0.0016	0.0017	0.0017	0.0017
S(D,$J_{\text{cell,cell}}$)	0.0127	0.0127	0.0127	0.0126
S(λ_A,$J_{\text{cell,cell}}$)	0.0102	0.0102	0.0102	0.0101
S(λ_c,D,λ_A)	0.0098	0.0102	0.0104	0.0107
S(λ_c,D,$J_{\text{cell,cell}}$)	0.0253	0.0257	0.0262	0.0268
S(λ_c,λ_A,$J_{\text{cell,cell}}$)	0.0214	0.0217	0.0220	0.0225
S(D,λ_A,$J_{\text{cell,cell}}$)	0.0073	0.0075	0.0076	0.0078
