# Supplementary material for: A global sensitivity analysis approach for morphogenesis models
Source: BMC Syst Biol. 2015 Nov 21;9:85. doi: 10.1186/s12918-015-0222-7 (PMC4654849; doi:10.1186/s12918-015-0222-7)
Supplement: Additional file 3 — Table S2. Global sensitivity analysis results for lacuna count. (PDF 51.5 Kb) [file 12918_2015_222_MOESM3_ESM.pdf]

Table S2 Global sensitivity analysis results for lacuna count.

$\hat{p}$	11	12	13
Variance data	15.8651	15.8651	15.8651
Variance PCE	15.5295	15.9661	16.7974
S(λ_c)	0.0075	0.0074	0.0070
S(D)	0.7120	0.7130	0.7187
S(λ_A)	0.0129	0.0125	0.0119
S($J_{\mathrm{cell,cell}}$)	0.0418	0.0407	0.0387
S(λ_c,D)	0.0579	0.0570	0.0552
S(λ_c,λ_A)	0.0043	0.0043	0.0041
S(λ_c,$J_{\mathrm{cell,cell}}$)	0.0144	0.0145	0.0143
S(D,λ_A)	0.0350	0.0347	0.0348
S(D,$J_{\mathrm{cell,cell}}$)	0.0533	0.0521	0.0501
S(λ_A,$J_{\mathrm{cell,cell}}$)	0.0050	0.0048	0.0046
S(λ_c,D,λ_A)	0.0289	0.0315	0.0331
S(λ_c,D,$J_{\mathrm{cell,cell}}$)	0.0222	0.0232	0.0236
S(λ_c,λ_A,$J_{\mathrm{cell,cell}}$)	0.0118	0.0131	0.0142
S(D,λ_A,$J_{\mathrm{cell,cell}}$)	0.0086	0.0094	0.0100
