# Supplementary material for: A global sensitivity analysis approach for morphogenesis models
Source: BMC Syst Biol. 2015 Nov 21;9:85. doi: 10.1186/s12918-015-0222-7 (PMC4654849; doi:10.1186/s12918-015-0222-7)
Supplement: Additional file 4 — Table S3. Global sensitivity analysis results for lacuna count capped at maximum of 5 lacunae. (PDF 52.3 Kb) [file 12918_2015_222_MOESM4_ESM.pdf]

Table S3 Global sensitivity analysis results for lacuna count capped at maximum of 5 lacunae.

$\hat{p}$	12	13	14
Variance data	3.4870	3.4870	3.4870
Variance PCE	3.4346	3.4821	3.5668
S(λ_c)	0.0081	0.0081	0.0079
S(D)	0.6893	0.6861	0.6847
S(λ_A)	0.0078	0.0077	0.0077
S($J_{\text{cell,cell}}$)	0.0578	0.0571	0.0557
S(λ_c,D)	0.0561	0.0557	0.0547
S(λ_c,λ_A)	0.0084	0.0085	0.0086
S(λ_c,$J_{\text{cell,cell}}$)	0.0394	0.0409	0.0429
S(D,λ_A)	0.0048	0.0048	0.0048
S(D,$J_{\text{cell,cell}}$)	0.0489	0.0485	0.0476
S(λ_A,$J_{\text{cell,cell}}$)	0.0035	0.0035	0.0035
S(λ_c,D,λ_A)	0.0147	0.0160	0.0169
S(λ_c,D,$J_{\text{cell,cell}}$)	0.0458	0.0476	0.0490
S(λ_c,λ_A,$J_{\text{cell,cell}}$)	0.0239	0.0258	0.0274
S(D,λ_A,$J_{\text{cell,cell}}$)	0.0121	0.0132	0.0140
